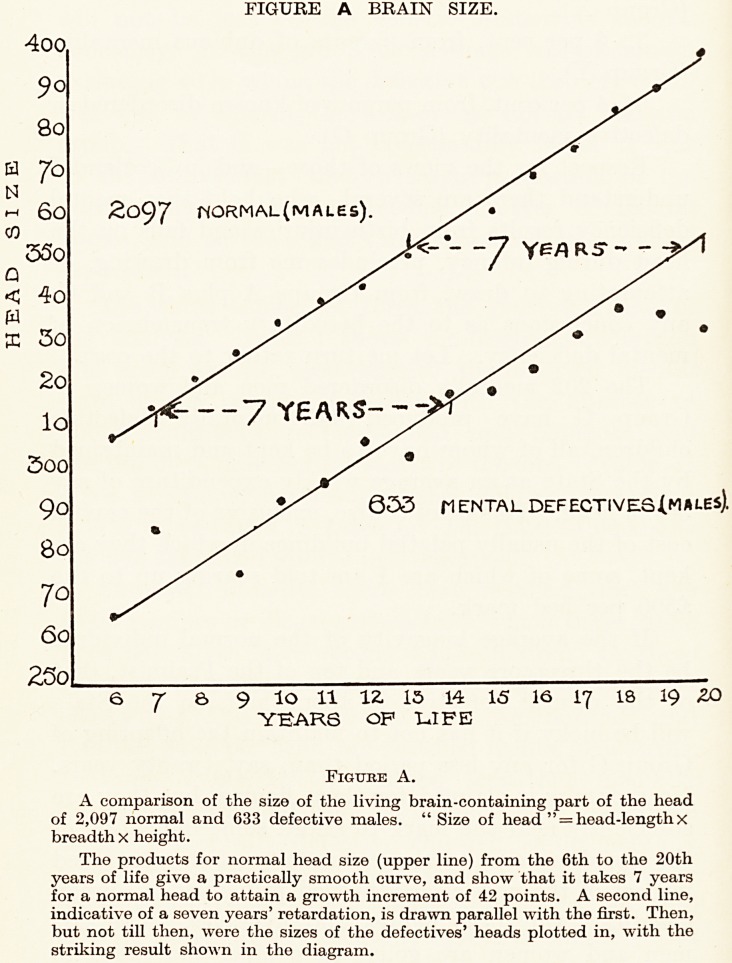# An Investigation into the Mental State of the Parents and Sibs of 1,050 Mentally Defective Persons
*A paper read at the Seventh International Genetical Congress, Edinburgh August, 1939.


**Published:** 1939

**Authors:** Richard J. A. Berry

**Affiliations:** Director of Medical Services, Stoke Park Colony, Bristol; Chairman of the Burden Mental Research Trust


### AN INVESTIGATION INTO THE MENTAL STATE OF THE PARENTS AND SIBS OF 1,050 MENTALLY DEFECTIVE PERSONS.*

BY

RICHARD J. A. BERRY, M.D., F.R.C.S., F.R.S.E.,

*Director of Medical Services, Stoke Park Colony, Bristol; Chairman of the Burden Mental Research Trust.*

---

To the title of this paper there should properly be added the words "based on second-hand information," for the mental states of the parents are known to me only through the reports of those doctors and official visitors whose duty it is to make them.

Dr. L. S. Penrose's *Clinical and Genetic Study of 1,280 Cases of Mental Defect,* published in 1938, was free from these difficulties, hence his analytical methods were different. Even if they had not been, the results would still not have been strictly comparable. Penrose himself says that "great care is necessary when comparing the results of one study on mental deficiency with those of another, for in practice both are beset by formidable difficulties." Formidable indeed have been those of the present analysis, so without attempting any comparisons let it suffice to state the facts elicited.

Of the 1,050 certified mental defectives whose parentage forms the subject of this paper, all are, or have been, inmates of Stoke Park Colony, Bristol. They are drawn from thirty-eight different counties of

*A paper read at the Seventh International Genetical Congress, Edinburgh August, 1939.

England and Wales, and were diagnosed after admission as follows :—

| | |
|---|---|
| Idiots .. .. .. .. | 235 |
| Imbeciles .. .. .. | 500 |
| Feeble-minded .. .. | 243 |
| Mongol idiots .. .. | 42 |
| Mongol imbeciles .. .. | 30 |
| | 1,050 |

For all of these there is available full and accurate first-hand information of their mental, physical, medical and neurological states, but with their parents it is otherwise.

For any knowledge of the mental status of the parents we are dependent on second-hand data, not always reliable, sometimes insufficient, and only too frequently just simply given as " not known " or " not stated." A similar lack of detailed information also applies to many of the sibs. If this information were returned, as it ought to be, in a much less slip-shod manner, it might eventually become a fairly useful weapon in the secondary armaments of the geneticist. All these disabilities notwithstanding, there have accrued from an analysis of the available data some interesting and suggestive results.

In the official returns of the mental states of the parents of these 1,050 defective children some are stated to be both mentally normal. Others are returned as one parent mentally normal, and the other not known, not stated, dead or disappeared—and such piebald descriptions do little or nothing to soothe the mind of the curiously inquiring biologist.

In another group of parents, the information is devastating in its accuracy, as it is capable of proof and definitely points to profound mental disorder in the parents.

Between these two came larger and more indeterminate groups of parents whose mentality was not known, not observed, dubious, or unobtainable: the last owing to the fact that the defective child was illegitimate, and one or other of the parents, usually the father, had disappeared, to repopulate the world with other illegitimates by other mates.

Notwithstanding that we commenced the analysis by a subdivision of the parents into the several mental groups described by the visitors (Table I), it soon became obvious that no good purpose would be served by so continuing. On the contrary, the five groups of parents of Table III seemed to emerge, as follows:—

First—Group A. Where both parents and all sibs were of known mental normality.

Second — Group B. Both parents said to be mentally normal, but with sibs of unknown or proved defective mentality.

Third—Group X. One or other of the two parents of normal, the other of unknown, mentality, and with sibs of normal, unknown, or defective mentality.

Fourth—Group Y. Both parents of unknown, and / or with blood relations of dubious, mentality.

Fifth—Group G. One or both parents of known disordered mentality.

In Group B of Tables I and III there were 111 families where all the parents were mentally normal and the sibs of defective or unknown mentality. Of the 555 children from this parentage 3·2 per cent. were of normal mentality, 24·1 per cent. mentally defective, and the remaining 72·6 per cent. not stated.

In Group X of Table III (C and D of Table I) the mother was of known normal mentality in 48 instances, and the father in 64, that of the other parent being unknown. These 112 families produced 396 children, of whom 45 per cent. were of unstated mentality, 23·2 per cent. of normal, and 31·8 per cent. of defective mentality.

Group Y of Table III (E and F of Table I) is a large, heterogeneous, and mentally suspicious group. It comprises 462 families—of which 70 are illegal unions, the children illegitimate and the parents have disappeared. Of the remaining 392 families of legitimate parentage 151 have had parents and/or blood relatives of very suspicious mentality and of dubious genetic stock. They were variously described as "mentally nervous," "hysterical," "alcoholic," "suicidal," and with insane or mentally defective relatives. The father, for example, may have had some mentally defective nephews or nieces, or the mother may have had some cousins in a mental home. All these were rigorously excluded from Group G, where only positive evidence of the abnormal mental state of the parents was admitted. In the remaining 241 families of the Group, the mentality of both parents was unknown.

As thus composed, the 462 families of Group Y (Table III), with parents of unknown or very dubious mentality, have produced 1,737 children including the illegitimates. Of these 567 are mentally defective, 1,027 are of unknown mentality, and only 143 are definitely stated to have been mentally normal. The corresponding percentages are 32·6, 59·2 and 8·2.

We turn next to Groups A alone or A plus B and G (Table III) which afford the most dramatic genetic contrast of all. In Group A alone there are 163 families where all the parents are of known normal mentality. In Group G there are 202 families with parents of known grossly disordered mentality, as follows :—

Mothers :—

61 certified mental defectives.
16 certified insane.
44 of very low mentality.

Fathers :—

8 certified mental defectives.
7 certified insane.
14 of very low mentality.

Both parents :—

2 mentally defective.
50 of very low mentality.

In 24 of the 163 families with normal parents and normal sibs the defective was the only child. From these families there resulted 613 children, 163 of which were mentally defective in the proportions of 66 idiots, 68 imbeciles, 8 feeble-minded, and 21 Mongols. The remaining 450 children were mentally normal. Expressed in percentages these mentally normal parents produced 73·4 per cent. normal children, and 26·6 per cent. defective, with an average-sized family, excluding the 24 with an only child, of 4·2 children, one of whom was a defective.

Turning next to the 202 families of the defective-demented parents of Group G, there is a startling contrast. The group produced 814 children, of whom more than one-half, 54·4 per cent., are mentally defective, 4·5 per cent. described as mentally normal, and the remainder not stated.

Of the 202 children of this group who became inmates of Stoke Park, 27 were idiots, 110 imbeciles, 61 feeble-minded, and 4 Mongols. The significance of the high proportion of feeble-minded and low incidence of Mongols in this group as compared with Group A will not be lost on the genetic student of mental deficiency. In this group the average size of family, excluding 40 in whom the defective was the only child, was 4·8, as against the 4·2 of the normal parental group. That it was not considerably larger is due to the simple fact that a large proportion of the parents have themselves become inmates of mental homes, and so have had their reproductive activities suppressed.

The three tables set forth the general results of the analysis. Table I shows the original subdivision into seven groups, according to the reported mental state of the parents, together with the number of idiots, imbeciles, feeble-minded and Mongols, and the numbers and mental states of the sibs. Table II gives a summarized result of the children, defectives and sibs, recorded in Table I. The third table shows the five simplified groups just described.

Surveyed broadly, there have resulted from the 1,050 families or illegal unions from which these Stoke Park defectives have sprung 4,115 children. Of these 1,942 or 47·2 per cent. are of unknown or unstated mentality; 740 or 18·0 per cent. are normal; and 1,433 or 34·8 per cent. are certified mental defectives.

From Table III it will be seen that mentally disordered parents (Group G) produce more than twice as many mentally defective children as do mentally normal parents, even though some of the latter may have been carriers. It is further of interest to note that the percentage of mentally defective children rises with the increasing doubts of the parents' mental stability. Thus :—

25·4 per cent. defectives from the normal parents of Groups A and B.

31·8 per cent. from parents of unknown mentality (Group X).

32·6 per cent. from parents of dubious mentality (Group Y).

54·4 per cent. from parents of known disordered or defective mentality (Group G).

Respect for the views of those—and in Scotland I understand there are several—who hold that mental deficiency results from birth injuries and falls on the head during infancy, precludes me from drawing, or attempting to draw, from Groups A plus B and G, any conclusions as to the hereditary transmission of mental deficiency. Let me turn rather to the cost.

The 202 mentally disordered men and women of Group G have produced 443 mentally defective children, all of whom have to be kept and maintained by the State at an average weekly expenditure of say 30s. a head. This is, of course, exclusive of the capital cost of the usually palatial buildings in which they are kept, some of which are I am told soaring up to the £500 per bed mark.

If the average longevity of the normal individual be the threescore years and ten of the Psalmist, that of the mental defective is about half, so the State will be lucky if it has not to maintain the offspring of Group G for any less period than, say, twenty years. No accuracy is claimed for these figures, but they are sufficiently near the mark to afford some indication of the appalling cost to the community of its continued neglect of the now clearly proved principles of human genetics. On these figures and averages, these 202 men and women are going to compel the State to expend over half a million pounds on the maintenance of their mentally derelict offspring. Many of the parents themselves are also being fed, clothed and housed at

FIGURE **A** BRAIN SIZE.

FIGURE A.

A comparison of the size of the living brain-containing part of the head of 2,097 normal and 633 defective males. "Size of head" = head-length × breadth × height.

The products for normal head size (upper line) from the 6th to the 20th years of life give a practically smooth curve, and show that it takes 7 years for a normal head to attain a growth increment of 42 points. A second line, indicative of a seven years' retardation, is drawn parallel with the first. Then, but not till then, were the sizes of the defectives' heads plotted in, with the striking result shown in the diagram.

the State's expense, but even this does not complete the gravity of the social menace.

In February of this year I published in the *British Medical Journal* a short paper on the "Social Aspects of Mental Deficiency in the Wage-earning Classes." It was there pointed out that in 31·5 per cent. of the feeble-minded families there and here dealt with, one or more of their members had been actively recruiting the criminal classes, so that the ultimate final cost to the community of the irresponsible sexual and anti-social activities of the members of Group G may well be nearer the million mark than the half-million. In either case the cost seems to be out of all proportion to their worth.

Assuming, for the moment and in conclusion, that the significant difference in the percentage of defective children from normal and defective parents—54·4 per cent. in the latter as against the 25·4 per cent. of Groups A and B together, really is due to genetic factors, that is, is of hereditary origin, what is it that is inherited? It is certainly not, as is often assumed, a mental state. A mental state is no more inherited than is speech. Man is born not with the power of speech, but only with the nerve potentialities for its acquisition. Nor is he born with a mental state, but only with a brain for the ultimate development of that state. It can now be definitely accepted that mental deficiency usually results from a brain whose growth has become arrested, impaired, or damaged at some time prior to birth (see Table IV). Lack of growth (see Figure A) is indeed a characteristic of all defectives, and that growth-lack shows itself in their small heads and stature, their deficient weight, their small brain—in fact, in practically all parts of their undeveloped bodies except, unfortunately, their reproductive organs.

May, therefore, a biologist conclude by suggesting that what are inherited are altered potentialities for growth and not specific mental states ?

TABLE I.

SHEWING SEVEN GROUPS, WITH THE DIAGNOSIS AND NUMBERS OF THE MENTALLY DEFECTIVE PATIENTS, AND THE NUMBERS OF SIBS OF KNOWN AND UNKNOWN MENTALITY.

A. 163 *patients with mentally normal parents and normal sibs* (1 *defective child only*).

| Nos. | Diagnosis. | No Sibs. | Normal Sibs. | Unknown Mentality. | Defective Sibs. | Sibs. |
|---|---|---|---|---|---|---|
| 66 | Idiots .. .. | 11 | 160 | 0 | 0 | 160 |
| 68 | Imbeciles .. .. | 11 | 191 | 0 | 0 | 191 |
| 8 | Feeble-minded .. | 0 | 34 | 0 | 0 | 34 |
| 21 | Mongols .. .. | 2 | 65 | 0 | 0 | 65 |
| 163 | | 24 | 450 | 0 | 0 | 450 |

B. 111 *patients with mentally normal parents and with sibs of defective or unknown mentality.*

| | | | | | | |
|---|---|---|---|---|---|---|
| 35 | Idiots .. .. | 0 | 3 | 112 | 8 | 123 |
| 44 | Imbeciles .. .. | 0 | 12 | 147 | 5 | 164 |
| 21 | Feeble-minded .. | 0 | 3 | 105 | 9 | 117 |
| 11 | Mongols .. .. | 0 | 0 | 39 | 1 | 40 |
| 111 | | 0 | 18 | 403 | 23 | 444 |

C. 48 *patients. Mother mentally normal. Father unknown.*

| | | | | | | |
|---|---|---|---|---|---|---|
| 3 | Idiots .. .. | 0 | 7 | 7 | 0 | 14 |
| 26 | Imbeciles .. .. | 8 | 23 | 34 | 5 | 62 |
| 18 | Feeble-minded .. | 12 | 14 | 9 | 0 | 23 |
| 1 | Mongols .. .. | 1 | 0 | 0 | 0 | 0 |
| 48 | | 21 | 44 | 50 | 5 | 99 |

D. 64 *patients. Father mentally normal. Mother unknown.*

| | | | | | | |
|---|---|---|---|---|---|---|
| 11 | Idiots .. .. | 3 | 7 | 25 | 0 | 32 |
| 30 | Imbeciles .. .. | 6 | 29 | 60 | 4 | 93 |
| 16 | Feeble-minded .. | 3 | 4 | 36 | 5 | 45 |
| 7 | Mongols .. .. | 1 | 8 | 7 | 0 | 15 |
| 64 | | 13 | 48 | 128 | 9 | 185 |

E. 311 *patients with mentality of both parents unknown* (241 *legitimate*, 70 *illegitimate*).

| Nos. | Diagnosis. | No Sibs. | Normal Sibs. | Unknown Mentality. | Defective Sibs. | Sibs. |
|---|---|---|---|---|---|---|
| 63 | Idiots .. .. | 16 | 14 | 162 | 8 | 184 |
| 151 | Imbeciles .. .. | 59 | 9 | 341 | 31 | 381 |
| 82 | Feeble-minded .. | 41 | 3 | 104 | 18 | 125 |
| 15 | Mongols .. .. | 4 | 0 | 39 | 1 | 40 |
| 311 | | 120 | 26 | 646 | 58 | 730 |

F. 151 *patients with parents and/or blood relatives of very suspicious mentality.*

| | | | | | | |
|---|---|---|---|---|---|---|
| 30 | Idiots .. .. | 4 | 39 | 43 | 10 | 92 |
| 71 | Imbeciles .. .. | 17 | 64 | 178 | 29 | 271 |
| 37 | Feeble-minded .. | 5 | 7 | 119 | 8 | 134 |
| 13 | Mongols .. .. | 3 | 7 | 41 | 0 | 48 |
| 151 | | 29 | 117 | 381 | 47 | 545 |

G. 202 *patients with parents of known disordered mentality.*

| | | | | | | |
|---|---|---|---|---|---|---|
| 27 | Idiots.. .. .. | 7 | 6 | 41 | 58 | 105 |
| 110 | Imbeciles .. .. | 21 | 18 | 174 | 120 | 312 |
| 61 | Feeble-minded .. | 12 | 13 | 104 | 62 | 179 |
| 4 | Mongols .. .. | 0 | 0 | 15 | 1 | 16 |
| 202 | | 40 | 37 | 334 | 241 | 612 |
| 1050 | | 247 | 740 | 1942 | 383 | 3065 |

### TABLE II.

THE SAME SEVEN GROUPS AS IN TABLE I WITH THE NUMBERS AND PERCENTAGES OF CHILDREN OF KNOWN, UNKNOWN, AND DEFECTIVE MENTALITY.

| Group. | Nos. of Children. | Nos. of Unknown Children. | Nos. of Normal Children. | Nos. of Defective Children. | Percent-age of Unknown Children. | Percent-age of Normal Children. | Percent-age of Defective Children. |
|---|---|---|---|---|---|---|---|
| A | 613 | 0 | 450 | 163 | 0 | 73·4 | 26·6 |
| B | 555 | 403 | 18 | 134 | 72·6 | 3·2 | 24·1 |
| C | 147 | 50 | 44 | 53 | 34·0 | 29·9 | 36·1 |
| D | 249 | 128 | 48 | 73 | 51·4 | 19·3 | 29·3 |
| E | 1,041 | 646 | 26 | 369 | 62·1 | 2·5 | 35·5 |
| F | 696 | 381 | 117 | 198 | 54·0 | 16·8 | 28·5 |
| G | 814 | 334 | 37 | 443 | 41·1 | 4·5 | 54·4 |
| Totals .. | 4,115 | 1,942 | 740 | 1,433 | 47·2 | 18·0 | 34·8 |

### TABLE III.

SHOWING THE SEVEN GROUPS OF TABLES I AND II REDUCED TO THE FIVE SIMPLIFIED ONES OF THE TEXT, WITH THE NUMBERS AND PERCENTAGES OF THE CHILDREN OF KNOWN, UNKNOWN AND DEFECTIVE MENTALITY.

| Group. | Nos. of Children. | Nos. of Unknown Children. | Nos. of Normal Children. | Nos. of Defective Children. | Percentage of Unknown Children. | Percentage of Normal Children. | Percentage of Defective Children. |
|---|---|---|---|---|---|---|---|
| A .. .. | 613 | 0 | 450 | 163 | 0 | 73·4 | 26·6 |
| B .. .. | 555 | 403 | 18 | 134 | 72·6 | 3·2 | 24·1 |
| X=C & D of Tables I and II .. | 396 | 178 | 92 | 126 | 45 | 23·2 | 31·8 |
| Y=E & F of Tables I and II .. | 1,737 | 1,027 | 143 | 567 | 59·2 | 8·2 | 32·6 |
| G .. | 814 | 334 | 37 | 443 | 41·1 | 4·5 | 54·4 |
| Totals .. | 4,115 | 1,942 | 740 | 1,433 | 47·2 | 18·0 | 34·8 |

Groups A and B combined afford a more correct genetic contrast with Group G than does A alone. In this case the percentage of defective children from the former is 25·4 per cent as against the 54·4 per cent. of the latter.

### TABLE IV.

BRAIN-WEIGHT.

Weights of stripped and fluid-free right cerebral hemispheres of 150 ADULT defectives compared with weights of those of NORMAL CHILDREN below eight years of age. The third column shows the *percentage* of defectives with hemispheres of the weights shewn in the second.

| Age of Normal Child in years. | Weight of hemisphere of Normal Child, in grams. | Percentage of Adult Defectives with hemispheres of same weight. | |
|---|---|---|---|
| 1 | 380 | 17·6 | 84·6 per cent. at or below normal weight for four years old. |
| 2 | 448 | 25·3 | |
| 3 | 490 | 23·4 | |
| 4 | 520 | 18·2 | |
| 5 | 530 | 5·1 | 9·3 per cent. normal or subnormal weight. |
| 6 | 540 | 1·4 | |
| 7 | 550 | 1·4 | |
| 8 | 560 | 1·4 | |
| Overweight | | 6·1 | 6·1 per cent. pathological overweight. |

25 per cent. of defectives have hemispheres of weight of that of normal two-year-old infant. Only 15·4 per cent. have hemispheres weighing more than that of normal four-year-old, and of these 6·1 per cent. owe weight to pathological conditions.

## Figures and Tables

**Figure A. f1:**